# Oral and Gut Microbial Dysbiosis and Non-alcoholic Fatty Liver Disease: The Central Role of *Porphyromonas gingivalis*

**DOI:** 10.3389/fmed.2022.822190

**Published:** 2022-03-02

**Authors:** Ting Wang, Taichi Ishikawa, Minoru Sasaki, Toshimi Chiba

**Affiliations:** ^1^Division of Internal Medicine, Department of Oral Medicine, Iwate Medical University, Morioka, Japan; ^2^Division of Molecular Microbiology, Department of Microbiology, Iwate Medical University, Morioka, Japan

**Keywords:** oral microbiota, gut microbiota, microbial dysbiosis, NAFLD, *P. gingivalis*

## Abstract

Gut microbiota play many important roles, such as the regulation of immunity and barrier function in the intestine, and are crucial for maintaining homeostasis in living organisms. The disruption in microbiota is called dysbiosis, which has been associated with various chronic inflammatory conditions, food allergies, colorectal cancer, etc. The gut microbiota is also affected by several other factors such as diet, antibiotics and other medications, or bacterial and viral infections. Moreover, there are some reports on the oral-gut-liver axis indicating that the disruption of oral microbiota affects the intestinal biota. Non-alcoholic fatty liver disease (NAFLD) is one of the systemic diseases caused due to the dysregulation of the oral-gut-liver axis. NAFLD is the most common liver disease reported in the developed countries. It includes liver damage ranging from simple steatosis to nonalcoholic steatohepatitis (NASH), cirrhosis, and cancer. Recently, accumulating evidence supports an association between NAFLD and dysbiosis of oral and gut microbiota. Periodontopathic bacteria, especially *Porphyromonas gingivalis*, have been correlated with the pathogenesis and development of NAFLD based on the clinical and basic research, and immunology. *P. gingivalis* was detected in the liver, and lipopolysaccharide from this bacteria has been shown to be involved in the progression of NAFLD, thereby indicating a direct role of *P. gingivalis* in NAFLD. Moreover, *P. gingivalis* induces dysbiosis of gut microbiota, which promotes the progression of NAFLD, through disrupting both metabolic and immunologic pathways. Here, we review the roles of microbial dysbiosis in NAFLD. Focusing on *P. gingivalis*, we evaluate and summarize the most recent advances in our understanding of the relationship between oral-gut microbiome symbiosis and the pathogenesis and progression of non-alcoholic fatty liver disease, as well as discuss novel strategies targeting both *P. gingivalis* and microbial dysbiosis.

## Introduction

Alteration of the normal microbial composition, known as microbial dysbiosis, has been widely studied owing to its pathologic impacts on the body. Numerous studies have provided evidence that gut dysbiosis is closely related to systemic diseases centered in various organs (1). Moreover, recent studies have revealed that periodontal disease, which is a typical dysbiosis disease of the oral cavity, also contributes to the pathogenesis of systemic diseases, such as metabolic syndrome, cardiovascular disease, kidney disease, and brain disease, and may be related to cancer (2, 3). The pathogenic mechanisms underlying the effect of microbial dysbiosis beyond its original location are complicated, including direct effects of pathologic bacteria translocated by the blood to a specific organ, and effects of bacteria-derived endotoxins, metabolites, and inflammatory immune-mediators (4–6). Specifically, dysbiosis of the oral microbiota can be pathogenic to the internal organs *via* gut dysbiosis (7).

**Figure 1:**
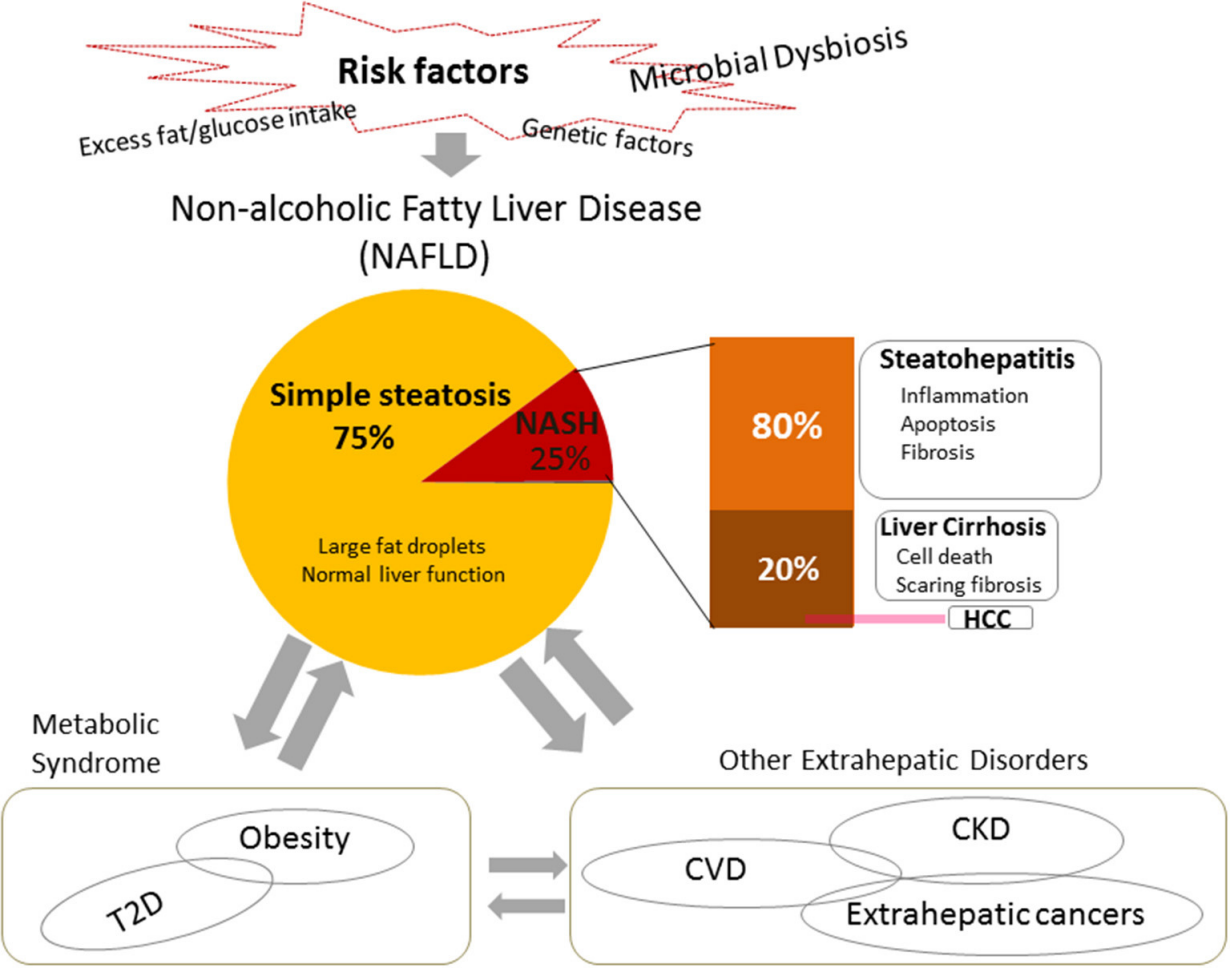
Risk factors, pathogenesis, and systemic impacts of non-alcoholic fatty liver disease (NAFLD). NAFLD is the most common chronic liver disease worldwide, usually presenting as fatty hepatocytes (simple steatosis). Non-alcoholic steatohepatitis (NASH) is a severe type of NAFLD. It is usually characterized by inflammation, cell damage, and apoptosis, and may include fibrosis. In turn, it can progress to cirrhosis or end-stage liver disease, i.e., hepatocellular carcinoma (HCC). In addition to heredity, one of the most widely accepted risk factors for NAFLD is excessive fat/glucose intake. Recent studies indicate that microbial dysbiosis takes an important role in the pathogenesis and the development of NAFLD. NAFLD leads not only to the development of hepatic diseases, but also connects with and contributes to systemic diseases, such as metabolic syndrome and extrahepatic disorders. NASH, non-alcoholic steatohepatitis; HCC, hepatocellular carcinoma; CDVs, cardiovascular diseases; CKD, chronic kidney disease, T2D, type 2 diabetes.

Non-alcoholic fatty liver disease (NAFLD) is of interest because, as is typical of metabolic diseases closely related to metabolic syndromes such as obesity and type 2 diabetes, it is also a systemic disorder affecting not only the liver but also various extrahepatic organs throughout the body and is linked to various systemic diseases, such as cardiovascular complications, kidney disease, and in particular the increase of extrahepatic malignancies (8, 9) (Figure 1). Studies on NAFLD have been limited because of its unclear origin, pathogenesis, and development, difficulties in early diagnosis, and a lack of effective therapeutic options. In this review, we focus on the relationship between NAFLD and *Porphyromonas gingivalis*, which is a typical oral pathogenic bacteria in periodontal disease (10, 11).

## Human-Microorganism Symbiosis

In recent years, PCR, quantitative PCR, and metagenomic analyses have been used to investigate indigenous bacterial populations more deeply than can conventional culture methods and microscopic observations, and have made great progress. As a result, it has been found that there are more than 1,000 types of microorganisms in the human body, and that they have, collectively, 3.3 million genes, far exceeding the 25,000 genes in the human genome (12). The total amount of genomic information possessed by microorganisms in a certain environment is called a microbiome; Lederberg has written that “human beings are super-living organisms composed of symbiotic microorganisms and human beings, and symbiotic microorganisms are extremely important to human beings” (13). Even sophisticated microbiota analysis can determine only the bacterial biota, whereas microbiome analysis can determine both the bacterial biota (number of bacteria, composition ratio) and its component functions and metabolism. Bacteria, fungi, and protozoa are among the microorganisms that are present throughout the human body. Typical indigenous fungi include *Candida albicans*, and protozoans include Trichomonas and amoeba*s* (14, 15). We focused on the bacteria that predominate among these indigenous microorganisms; they are present in healthy adults in or on the skin, nasopharynx, oral cavity, stomach, intestinal tract, vagina, etc. The number varies depending on the site, and the types vary from person to person (16–18). These bacteria are usually balanced to form a community (bacterial biota). Indigenous bacteria have beneficial effects on living organisms, such as acting against invasion and colonization by foreign pathogenic microorganisms, stimulating the immune system, enhancing host resistance and immune response, and synthesizing vitamins metabolites used by the living host body (19, 20). In contrast, such bacteria can also disadvantage the host by causing infectious disease in an easily infected host (opportunistic infection), or *via* pathogenic replacement, in which resident pathogenic microorganisms become predominant following reduction of the normal biota by antibacterial drugs. Moreover, resident bacteria that are not pathogenic in their original location can become pathogenic bacteria elsewhere (ectopic infection) (21). Indigenous bacteria are constantly exposed to the host's immune system, settle in the host tissues, and exist in a symbiotic, non-pathogenic state. It is thought that disruption of this interaction causes some infectious diseases (22).

## The Intestinal Biota

Indigenous bacterial communities in the intestinal tract are composed of various bacterial species, such as Firmicutes, Bacteroidetes, and Actinobacteria (23). These are thought to invade mainly through the oral cavity (24). To date, there have been numerous reports of microbiome analyses (Table 1).

**Table 1:** Reports of the human intestinal microbiome by human genome analysis.

**Published year**	**Participants**	**Country**	**The first author**	**PMID**
2016	106	Japan	Nishijima S	26951067
	1,135	Netherlands	Zhernakova A	27126040
2017	59	USA	Galloway-Peña J	28245856
	405	China	Jie Z	29018189
2018	106	China	Ye Z	30077182
	54	Nepal	Jha A	30439937
2019	267 pairs	Norway	Iszatt N	30813950
	2,170	USA	Shama A	31046835
2020	147	Cameroun	Lokmer A	32071424
	1,475	China	Xu F	33032658
2021	758	USA	Baniel A	33485388
	65	USA	Thapa S	33956889

Dysbiosis changes in the intestinal biota is closely related to various pathological conditions, including intestinal-related diseases and systemic diseases (25–28). For example, inflammatory bowel diseases, including Crohn's disease, have been associated with increased mucosa-associated facultive anaerobes, *Enterobacteriaceae, Pasteurellaceae, Veillonellaceae, Fusobacteriaceae*, and decreased *Faecalibacterium prausitzii, Erysipelotrichales, Bacteroidales*, and *Clostridiales* (26). In obese mouse model, an increase in Firmicutes proportion and a decrease in Bacteroidetes proportion have been confirmed (29). Furthermore, the proportion of *Akkermansia muciniphila*, of the Verrucomicrobia, was reduced in diabetic patients (30), and the feces of children with autism spectrum disorders contained nine unknown species of *Clostridium* (28).

## The Oral Biota

Previous reports suggest that the average adult has about 50–100 billion bacteria in the oral cavity, including as many as 500–700 bacterial species (31, 32). The oral biota varies across niches such as dental plaque, tongue, saliva, and gingival sulcus, and therefore exists as a wide variety of bacterial biota throughout the oral cavity (Table 2).

**Table 2:** Flora site and bacterial distribution in the human oral cavity^a^.

**Bacterial group**	**Bacterial distribution**			
	**Tooth surface (plaque)**	**Tongue**	**Saliva**	**Gingival crevice**
Gram-Positive facultative cocci	28.2	44.8	46.2	28.8
Streptococci	27.9	38.3	41.0	27.1
Staphylococci	0.3	6.5	4.0	1.7
Gram-Positive anaerobic cocci	12.6	4.2	13.0	7.4
Gram-Negative anaerobic cocci	6.4	16.0	15.9	10.7
Gram-Negative facultative cocci	0.4	3.4	1.2	0.4
Gram-Positive facultative rods	23.8	13.0	11.8	15.3
Gram-Positive anaerobic rods	18.4	8.2	4.8	20.2
Gram-Negative facultative rods	ND^b^	3.2	2.3	1.2
Gram-Negative anaerobic rods	10.4	8.2	4.8	16.1
Spirochetes	ND	ND	ND	1.0

a *Modified from Hamada and Slade (33)*.

b *ND, Not detected*.

While these oral bacteria play a role as indigenous bacteria, some are thought to cause local infections such as tooth decay, periodontal disease, and endodontic infections (34). In addition, recent oral microbiome analysis shows that not only some pathogenic bacteria induce local disease, but also an abnormal bacterial biota (dysbiosis), in which the oral biota is disturbed by the growth of certain keystone species, may be the cause of various oral diseases (35). In addition, many studies have reported that dysbiosis of the oral bacteria is associated with many systemic diseases, such as aspiration pneumonia, bacterial endocarditis, preterm birth, diabetes, Alzheimer's disease, and atherosclerosis (36–38). Oral streptococci, including *Streptococcus anginosus* and *S. intermedius*, which are gram-positive facultative anaerobic cocci, and *Prevotella, Fusobacterium*, and *Bacteroides*, which are gram-negative obligate anaerobic cocci, are listed as the causative bacteria of aspiration pneumonia (39). It has been reported that bacterial endocarditis has a very high isolation frequency of oral streptococci and these are considered a causative agent (40). These are referred to as ectopic infections. In contrast, in preterm birth, diabetes, Alzheimer's disease, atherosclerosis, and one of the autoimmune diseases, rheumatism, the involvement of gram-negative obligate anaerobic bacilli, such as *P. gingivalis* and *Treponema denticola*, which are classified as periodontal pathogens, is strongly suspected; however, it is rare that the causative organism is specifically detected in the lesion. Various studies have been undertaken into the pathogenic mechanism of these bacteria, but there are many unclear points (41–43).

## Mechanism by Which Oral Biota Induces Systemic Disease

Separate mechanisms underlying systemic disease caused by oral bacteria, differing from those involved in ectopic infection, have been considered. Conventionally, inflammatory cytokines and enzymes, present because of bacterial cell components in local lesions of the oral cavity, are transferred from the bloodstream to the whole body. More recently, it has been suggested that oral dysbiosis induces systemic disease by inducing intestinal dysbiosis, affecting the immune system, metabolic system, and intestinal barrier function (44, 45). Concerning this induction of intestinal dysbiosis by pre-existing oral dysbiosis, it has been reported that oral administration of *Fusobacterium nucleatum*, a gram-negative obligate anaerobic bacillus, significantly reduces the activity of natural killer cells (46). Additionally, *P. gingivalis*, whose proportion increases in periodontitis, may pass through the acid stomach and reach the intestines (47). In mice administered *P. gingivalis* orally, *P. gingivalis* does not colonize or proliferate in the intestinal tract, but changes the intestinal bacterial biota (48). The inside of the stomach is usually pH 1–2, and *P. gingivalis* cannot grow there. However, reports that *P. gingivalis* forms a biofilm and has a survival rate of 50% or more at pH 3, suggest that it can pass through the stomach at pH 4–5 immediately after eating (49). Changes in the intestinal biota may also decrease the expression of tight junction proteins involved in intestinal barrier function, increase inflammatory cytokine gene expression, and increase blood endotoxin levels because of suppression of the small intestinal alkaline phosphatase gene (50). As mentioned above, there are various reports on the mechanisms by which the oral biota can induce systemic diseases; further analysis is expected to contribute further details of these relationships, aiding the elucidation of the currently unclear etiology of systemic diseases.

## Fatty Liver and Non-Alcoholic Fatty Liver Disease

As blood circulation is the key pathway through which pathogenic bacteria and their toxic metabolites can reach distant locations across the whole body, it is reasonable that those organs with abundant blood supply are more vulnerable to the above substances, and more easily develop diseases related to oral and gut microbial dysbiosis. Among them, the liver is the largest solid internal organ and is unique in having two sources of blood supply: 80% from the portal vein and 20% from the hepatic artery. The liver plays critical roles in the body, such as regulating metabolic processes, including synthesizing glucose and lipids, maintaining homeostasis, and protecting against toxic substances by means of considerable detoxification abilities (9, 51).

The liver has the potential to regenerate following tissue damage, although chronic inflammatory damage or potent drug-induced toxicity may lead to various pathologies, including excessive fat accumulation (fatty liver), inflammation, fibrosis, and cirrhosis, or even to end-stage liver disease, hepatocellular carcinoma (HCC) (52, 53). Among these conditions, simple steatosis of hepatocytes only rarely leads to clinical symptoms; it is also difficult to make a diagnosis, as the only effective method of determination so far is biopsy, which is invasive and thus generally not acceptable to patients who are suspected of having such a disease. However, fatty liver may be a precursor to all of the other, more severe states of liver disease, even cancer (54, 55). Understanding the mechanisms underlying the etiology and pathology of fatty liver diseases therefore has definite clinical significance in prevention, early prognosis, and treatment. It is known that alcohol can lead to fatty liver (56), although fatty liver has also been found in non-drinkers. The latter is therefore called NAFLD.

NAFLD is currently regarded as the most common chronic liver disease worldwide (9). Data have shown that approximately one quarter of the world's population is affected by it (57). Approximately 25% of NAFLD patients develop a severe clinical phenotype called non-alcoholic steatohepatitis (NASH), which is characterized by inflammation, cell apoptosis, and fibrosis (58). The prevalence of NAFLD and NASH in adults in the United States ranges from 30–40% to 3–12%, respectively (59). Furthermore, ~20% of NASH patients may progress to cirrhosis (60), and NASH-associated cirrhosis increases the risk of hepatocellular carcinoma (HCC) by 2.4–12.8% (61, 62). The pathogenesis of NAFLD has not yet been completely elucidated. A “two-hit” theory has been widely supported (63). Firstly, excess food intake causes metabolic disorders such as insulin resistance, which lead to excess fat accumulation, simple steatosis, in the liver. Secondly, lipotoxicity causes oxidative stress, mitochondrial dysfunction, and endoplasmic reticulum stress, which induces cell injury, inflammation, and fibrosis. However, research over the last decade supports a new “multiple-hits” hypothesis. In addition to various dietary components and genetic factors, microbial dysbiosis is a crucial pathogenic factor and plays important roles in the development of NAFLD (8, 58) (Figure 1).

## Gut Microbial Dysbiosis and NAFLD: The Gut-Liver Axis

Risk factors such as long-term consumption of a high-fat diet, antibacterial drug use, and intestinal inflammation, cause alterations in gut microbial composition and functions (64–67). As a result, the gut barrier function is impaired, facilitating the entry of pathogenic bacteria, bacterial endotoxins, and other inflammatory cells and mediators to the portal vein, and thereby reaching the liver (62).

Numerous clinical and experimental studies have been conducted to reveal the relationships between gut dysbiosis and NAFLD, and the impact of gut dysbiosis on the etiology and pathology of NAFLD (65, 68, 69). Firstly, gut microbial alterations were detected in patients with NAFLD. For example, in the gut of the patients with NASH or cirrhosis, there was a decrease in health-related bacteria such as *Bacteroidetes* and an increase of pathogenic bacteria *Proteobacteria* and *Enterobacteriaceae* specie (70–72); The abnormal changes in the abundance of some bacterial phyla, such as *Bacteroides, Prevotella*, Proteobacteria, and Firmicutes, correlate with disease severity (73–76).

Secondly, microbiome signature profiles and metabolomics analysis have recently promoted wide discussion of the pathologic roles of bacteria-derived metabolites and products (77, 78). Such factors are known to contribute to hepatic steatosis, insulin resistance, and fibrosis (79–81). For example, the microbiome signature in NAFLD indicates that 3-(4-hydroxyphenyl), a metabolic material from *Firmicutes, Bacteroidetes* and Proteobacteria, is associated with liver fibrosis (82, 83), and Glycocholate is positively associated with advanced liver fibrosis (84). Moreover, metabolic studies have clarified that microbiome-generated secondary bile acid triggers NAFLD (85), and the gut microbiome of NAFLD patients shows abnormalities in carbon and amino acid metabolism (86), choline depletion, and increased production of certain short-chain fatty acids and alcohols (71). This suggests that gut dysbiosis is related to NAFLD by metabolic pathways.

Thirdly, endotoxemia resulted from increased gut permeability is also associated with NAFLD pathogenesis, indicating the effects of gut dysbiosis on immune system (55). Bacteria-derived endotoxins such as lipopolysaccharide (LPS) are closely related with intestinal immune function (73). Most reports give function of LPS as stimulation of the Toll-like receptor 4 (TLR4) (87, 88), which belongs to the Toll-like family of receptors that is crucial in host defense against invading pathogens (89). The localization of LPS has been confirmed in the livers of NALFD patients (58, 90, 91). Blocking LPS receptors is related to improved NAFLD in *in-vivo* studies (89, 92). Recent clinical and animal studies provide evidence that elevated concentrations of LPS and endotoxin-producing bacterial strains are positively related to the progression of NAFLD, *via* inflammation and oxidative stress, which finally leads to chronic inflammation (93) and insulin resistance (88, 94). LPS also triggers lipid peroxidation in the liver (95, 96). We will discuss LPS in the later part of this review.

It should be noted that although the above evidence indicates a promoter role for gut dysbiosis in the pathogenesis of NAFLD, the bilateral relationship between the disease and gut dysbiosis remains to be completely understood.

## *P. gingivalis* and NAFLD

Recently, interest has been shown in the pathogenic role of oral, as well as gut, microbial dysbiosis. Chronic oral diseases such as periodontal disease typically involve such dysbiosis. Multiple reports from epidemiologic, *in-vivo*, and *in-vitro* studies indicate that periodontal disease is closely associated with NAFLD (93, 97). Oral pathologic bacteria related to oral diseases include *P. gingivalis, Treponema denticola, Prevotella intermedia, Aggregatibacter actinomycetemcomitans, Tannerella forsythia*, and *Campylobacter rectus*. Among them, *P. gingivalis* is regarded as a key pathogen leading to periodontitis and related systemic diseases (3, 10, 98). In this review, we emphasize the relationship between *P. gingivalis* and NAFLD, the effects of *P. gingivalis* on the pathogenesis of NAFLD, the mechanism underlying its pathogenic functions, and the therapeutic approaches targeting *P. gingivalis* and microbial dysbiosis.

### *P. gingivalis-*Associated NAFLD

A potential link between *P. gingivalis* and NAFLD has been revealed. *P. gingivalis* and its DNA were detected in the oral cavities or livers of NAFLD patients at higher frequency than those of controls (99, 100). NASH patients with *P. gingivalis* infection had more severe fibrosis than those without infection (100). Furthermore, supportive data from various *in-vivo* studies showed that *P. gingivalis* infection can stimulate fat accumulation, increase the immune response, and result in insulin resistance, indicating the impact of *P. gingivalis* in NAFLD/NASH procession (97).

**Figure 2:**
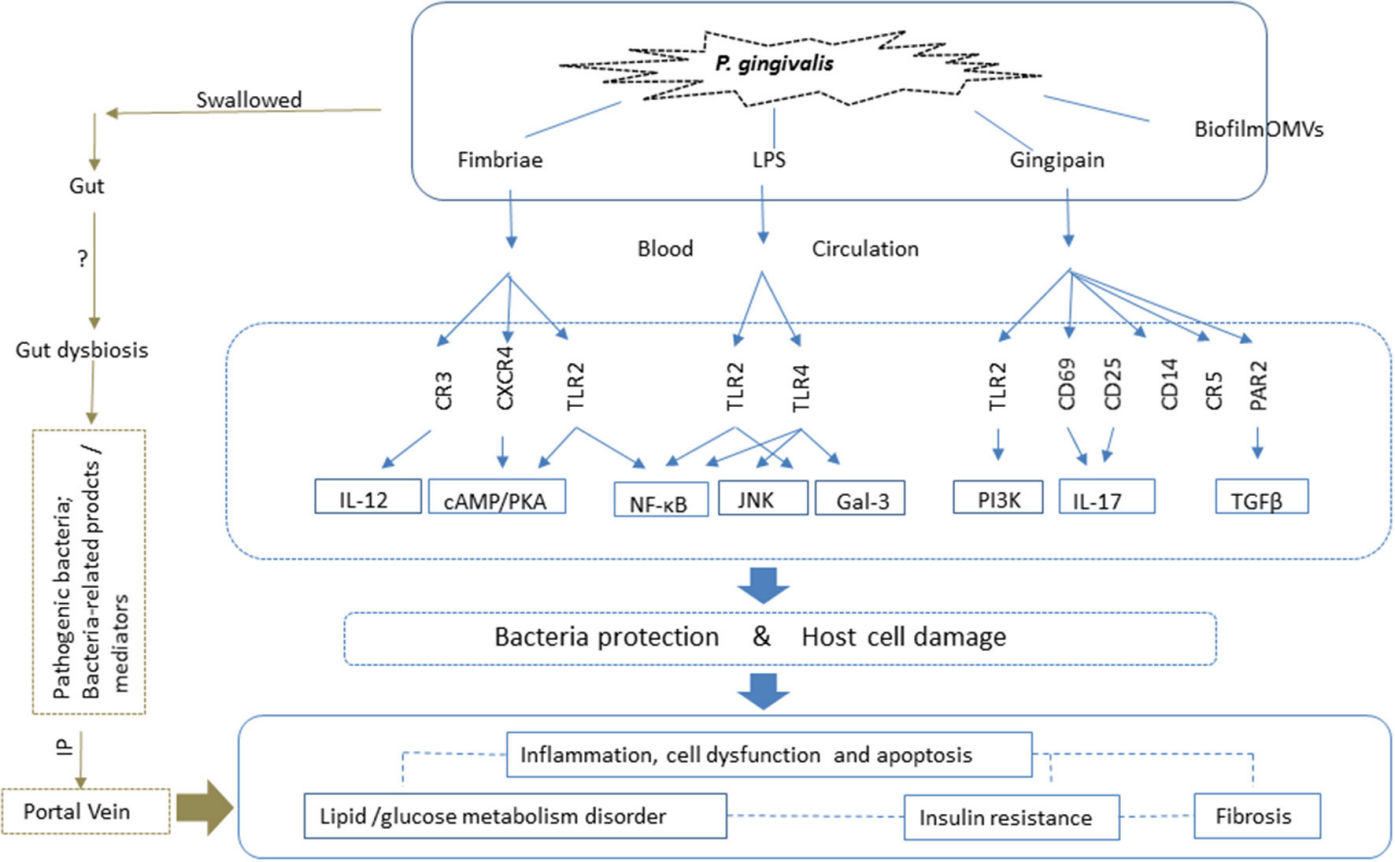
*P. gingivalis* impacts NAFLD *via* both the oral-liver and oral-gut axes. With respect to the oral-liver axis, *P. gingivalis* and its virulence factors can reach the liver directly *via* the blood circulation. Virulence factors such as fimbriae proteins, lipopolysaccharide (LPS) and gingipains elicit inflammatory immune reactions and activate intracellular signaling pathways by binding to the corresponding receptors. This results in inflammation, cell death, and dysfunctions, all of which are closely related to glucose and lipid metabolic disorders, insulin resistance, and fibrosis, which contribute to the pathogenesis of NAFLD. Biofilm and outer membrane vesicles (OMVs) play roles in the transfer of bacteria and virulence factors, such as gingipains, to the liver, the site of pathogenic action. *P. gingivalis* also affects NAFLD *via* the oral-gut-liver axis. It can enter the gastrointestinal tract *via* swallowing, where it can induce dysbiosis of the gut microbiota and enhance intestinal permeability. Thus, both the pathogenic bacteria, and related toxic products and mediators, can reach the liver *via* the portal vein, and play a role in NAFLD. LPS, lipopolysaccharide; TLR, Toll-like receptor; IL, interleukin; OMVs, outer membrane vesicles; CXCR, CXC-chemokine receptor; CR, complement receptor; cAMP, cyclic adenosine monophosphate; PKA, protein kinase A; NF-κB, nuclear factor kappa B; TGF-β, transforming growth factor β; PAR, proteinase-activated receptor; JNK, c-Jun-NH2-terminal kinase; IP, intestinal permeability; Gal, galectin; PI3K, phosphatidylinositol-3 kinase.

The spread of *P. gingivalis* to distant organs, such as the liver, occurs through two possible pathways. One is direct release into the blood circulation. During daily procedures such as brushing, or dental treatment, *P. gingivalis and* bacteria-associated factors such as LPS and cytokines spread into the blood *via* the microulceration in the periodontal pocket, and are transported to the liver through the hepatic artery. The indirect pathway involves swallowing *P. gingivalis*, which can translocate to the gastrointestinal tract and induce alteration of gut microbiota; this in turn negatively affects the liver through the portal vein system (93, 101). Therefore, the pathogenic action of the bacteria is related to both these pathways: directly through the oral-liver axis, and indirectly through the oral-gut-liver axis (Figure 2).

### Direct Impacts of *P. gingivalis* in NAFLD: Structure-Related Functions

*P. gingivalis* is a gram-negative, obligate anaerobic bacteria. It is a common component of subgingival microbiomes that can colonize oral epithelial cells (102). *P. gingivalis* has unique structural components, known as virulence factors. These include some bacteria's own structural components such as fimbriae and LPS, and secretory components such as gingipains, and play critical roles in the survival and spread of *P. gingivalis* through cellular colonization, along with its pathogenic functions, such as inducing host immune response and inflammatory reactions (98, 103).

*P. gingivalis* fimbriae are filamentous structures on the surface of the bacterium that enable bacterial binding to host cells and tissues, enhance bacterial motility and invasiveness, and contribute to biofilm formation (104). There are two forms of *P. gingivalis* fimbriae: FimA, and the less important minor fimbriae. Studies of NAFLD patients showed that the invasive type II FimA constituted half of the total fimbriae detected (99). Type IV FimA, however, is closely associated with advanced liver fibrosis (105).

The mechanism underlying the impact of FimA on host cells is primarily related to the activation of adhesion and immuno-inflammatory pathways *via* interactions with various host receptors, which facilitates bacterial colonization and leads to host cell inflammation (104, 106, 107). In addition, FimA can also help the bacteria survive in the host cell over extended periods by triggering the complement system to protect the bacteria from host immune clearance (104).

FimA acts through three major types of receptor. Regarding its inflammatory effects, FimA binds to Toll-like receptor (TLR) 2, which activates the nuclear factor kappa B (NF-κB) system to induce the production of various pro-inflammatory cytokines (108). TLR2 has been shown to be associated with the pathogenesis of NAFLD (109). FimA also triggers the innate immune system by binding to complement receptor (CR) 3 and stimulating macrophages/monocytes (10). As a result, FimA inhibits the production of interleukin (IL) (12), which is related to bacterial clearance (110), thus enhancing *P. gingivalis* survival. CR3 is expressed in the liver (111), and the activation level of the complement system is related to NAFLD severity (112), suggesting the involvement of *P. gingivalis* fimbriae in the pathogenesis of NAFLD *via* immune response induction.

Furthermore, FimA activates CXC-chemokine receptor 4 (CXCR4) and TLR2 to induce cyclic adenosine monophosphate-dependent protein kinase A signaling, which not only results in disruption of phagocytosis by macrophages (113), but also inhibits host immune clearance of *P. gingivalis* (114). Dysfunctions of both macrophages and CXCR4 have been reported to be associated with NAFLD (115, 116).

Beyond FimA, *P. gingivalis* minor fimbriae can also induce inflammatory reactions in macrophages (117), and are reported to induce phosphorylation of FOXO1 (118), which plays a role in both lipid and glucose metabolism (119), indicating that both FimA and minor fimbriae in *P. gingivalis* may be factors leading to liver injury by this species.

LPS is a component of the outer cell membranes of gram-negative bacteria (120, 121). It critically contributes to the pathogenicity of microbes, mainly through lipid A, which is the biologically active region of LPS (122). LPS is structurally different in different bacterial species due to variations of fatty acid acyl chain composition of lipid A (10). *P. gingivalis* LPS contains multiple forms of lipid A, and the structural differences in lipid A may explain why *P. gingivalis* LPS can initiate differential signaling pathways and immune responses (10, 123). In addition to participating in biofilm formation (124)*, P. gingivalis* LPS plays an important role in triggering host inflammatory responses *via* TLR activation; the specific type of TLR activation by LPS depends on the variation in the acylation of lipid A (10). However, it was recently reported that the activation of TLR2 by *P. gingivalis* is *via* other molecules on the surfaces of intact *P. gingivalis* cells rather than lipid A (125). Activation of TLR2 leads to an increase in the production of cytokines such as tumor necrosis factor α (TNF-α) and IL-6 by macrophages (126), while activation of TLR4 elevates the levels of IL-1β, IL-6, and IL-8 (127). In addition, NF-κB signaling pathway has also been reported to act downstream of TLRs, and to play a critical role in the inflammatory function of *P. gingivalis* LPS, including the production of cytokines (123). Several *in-vivo* and *in-vitro* studies have been performed to clarify the effects of *P. gingivalis* LPS on NAFLD. Injection of *P. gingivalis* LPS into the gingiva of animal models resulted in lipid deposition and inflammation in the liver (128–130), and *in-vitro* studies using HepG2 cells indicated that LPS may play a role in intracellular lipid accumulation and inflammation *via* both NF-κB and c-Jun-NH2-terminal kinase signaling (131). Moreover, *P. gingivalis* LPS can accelerate the progression of mild fatty liver to NASH (132), and studies using steatotic hepatocytes revealed that this activity may be related to increased TLR2 expression, inflammasome mRNA levels, and pro-inflammatory cytokines (100). Moreover, recent evidence shows that *P. gingivalis* LPS may contribute to hepatic fibrosis by activating hepatic stellate cells (HSCs); the mechanism involves triggering TLR4 to increase the production of galectin-3, which is critical for HSC activation (132). In addition, *P. gingivalis* LPS is possibly implicated in insulin resistance, either by stimulating the activation of pro-inflammatory cytokines such as TNF-α and IL-6, which play important roles in insulin resistance (133), or by directly inhibiting glucose incorporation into smooth muscle cells (134).

Kuraji et al. reported that *P. gingivalis* LPS accumulated predominantly in the liver, over other organs, and persisted in the livers of HFD-induced steatotic mice longer than in normal mouse liver. In addition, the diseased mice showed enhanced sensitivity to LPS and delayed clearance of LPS from the liver, indicating the potent role of *P. gingivalis* LPS in liver injury and NAFLD. The mechanism is possibly related to increased and activated hepatic macrophages (Kupffer cells) and TLR signaling (93).

As noted elsewhere in this review, gut bacteria-derived LPS also plays critical roles in the pathogenesis of NAFLD. The different functions and mechanisms involved in gut bacteria-derived LPS and *P. gingivalis* LPS have been investigated. For example, *P. gingivalis* LPS can activate both TLR2 and TLR4, whereas *Escherichia coli* LPS can only bind to TLR4 (135); although both LPS types activate TLR4, *P. gingivalis* LPS stimulates different pathways from those affected by *E. coli* LPS (136). Furthermore, *P. gingivalis* LPS shows a stronger ability to escape recognition by the host innate defense system than *E. coli* LPS (137), and *P. gingivalis* LPS induces more intracellular fat accumulation in HepG2 cells than *E. coli* LPS (131). In contrast to the substantial accumulated evidence that gut bacteria-derived LPS contributes to NAFLD by inducing oxidative stress (138), there is as yet little evidence (139) supporting direct oxidative stress stimulation by *P. gingivalis* LPS in NAFLD, which should be investigated further.

Gingipains are a family of secretory cysteine proteinases that are known to be the main virulence factors related to the pathogenicity of *P. gingivalis*. They consist of lysine and arginine gingipains (140, 141). As with fimbriae and LPS, gingipains also play roles in biofilm formation and produce immune-inflammatory responses by activating various immune cells (10, 142). Evidence shows that gingipains protect *P. gingivalis* from the host defense system in the following ways: they enhance the production of TNF-α by neutrophils by activating the TLR2/phosphatidylinositol-3 kinase (PI3K) pathway (143), thereby facilitating bacterial survival within host cells; they inhibit pathogen clearance by negatively regulating the production of neutrophil-derived molecules (144) and the macrophage immune receptor CD14 (145); and they promote the adaptability of *P. gingivalis* by triggering the complement system, for example by modulating the C5a receptor (C5aR) and its crosstalk with TLR2 signaling in macrophages (146).

Furthermore, gingipains may play a role in evading the host adaptive immune system by regulating T-cell immunity (147). Like LPS (148), they can induce the production by Th17 cells of the cell-specific cytokine, IL-17, by directly inducing the expression of CD69 and CD25 on T-cells (149). T-cell immunity, especially the Th17/IL-17 signaling pathway, plays a role in protecting pathogens and promoting inflammation, and was recently reported as important in the development of NAFLD (150–153). Moreover, recent work highlights a remarkable function of gingipains in NAFLD, revealing that they contribute to liver fibrosis by activating HSCs *via* the proteinase-activated receptor (PAR) 2 and TGF-β pathway (132). It should also be noted that gingipains can inactivate PI3K, protein kinase B (Akt), and Akt downstream proteins, including glycogen synthase kinase 3 (GSK3) and mammalian target of rapamycin (mTOR) (154). The PI3K/Akt signaling pathway plays multiple roles in various cell functions, including cell survival and glucose metabolism, and an *in-vitro* study has revealed that *P. gingivalis* suppresses glycogen synthesis in HepG2 cells by inhibiting the insulin receptor substrate 1/Akt/GSK3β pathway (155). This indicates that gingipains possibly participate in glucose metabolism impairment/insulin resistance, which needs to be investigated further.

Biofilms and outer membrane vesicles (OMVs) are two important microorganism-produced structures responsible for the survival, spread, and pathogenicity of microbes. Biofilms are composed of water, bacterial cells, and extracellular polymeric substances, which are responsible for microorganism protection and resistance to clinical treatments, such as antibiotics (156–158). *P. gingivalis* virulence factors, such as fimbriae, LPS, and gingipains, have been reported to contribute to biofilm formation, as mentioned previously, and the pathogenicity of *P. gingivalis* is enhanced by biofilm (159, 160). In addition to being a reservoir of pathogenic bacteria, biofilms also contribute to inflammation in many diseases, including inflammatory bowel disease and hepatobiliary carcinomas (161); however, the pathogenic effect of *P. gingivalis* biofilm, such as direct reactions of biofilm in NAFLD, remains unclear.

OMVs are small, spherical, bilayered membrane structures that are constantly released from the bacterial surface during growth. Each vesicle is composed of outer membrane proteins, lipoproteins, LPS, and some periplasmic components (162, 163). Like other bacteria such as *Francisella* and *Pseudomonas putida, P. gingivalis* can produce OMVs (164). *P. gingivalis* OMVs can concentrate virulence factors such as gingipains and LPS in the form of OMVs and discharge them to the environment to participate in bacteria-associated disorders (165, 166). *P. gingivalis* OMVs play roles in biofilm formation by binding to other periodontopathogens (167, 168), and facilitate bacterial adhesion and invasion in host cells, adaption to stress, and immune defense evasion (168). In addition to contributing to the destruction of periodontal tissues, *P. gingivalis* OMVs can migrate to the blood and play an important role in the pathogenesis of various systemic diseases, such as cardiovascular disease, rheumatoid arthritis, Alzheimer's disease, and carcinoma; the mechanism involved has been recently reviewed by Zhang et al. (169). Although the direct impact of *P. gingivalis* OMVs in NAFLD has not been completely elucidated, *P. gingivalis* OMVs carrying gingipains can transfer to the liver and impair hepatic glycogen synthesis in a mouse model; they can also inhibit insulin-induced Akt/GSK-3β signaling in a gingipain-dependent manner in HepG2 cells (170), indicating a potential role of these gingipain-carrying OMVs in the development of NAFLD through negative regulation of glucose metabolism and insulin sensitivity.

In summary, these bacteria-associated structures play roles in bacterial protection, as well as affecting the virulence of *P. gingivalis*, by activating various risk factors for NAFLD, such as fat accumulation, inflammation, insulin resistance/disturbance of glucose metabolism, and fibrosis. The mechanism involves immune cell-related inflammatory reactions and various intracellular signaling pathways (Figure 2). The scope of this review includes only the reported virulence mechanisms of *P. gingivalis* in relation to the pathogenesis of NAFLD; the functioning of these mechanisms beyond NAFLD and in other diseases also requires consideration. Moreover, other virulence factors, such as heat shock protein 60 (171), have been mentioned in relation to liver diseases (172). However, the majority of *in-vitro* studies have focused on circulating immune cells, while few studies have investigated the role of *P. gingivalis* in hepatic cells such as Kupffer cells and hepatocytes. Further studies are needed to elucidate the roles of the pathogenic components of *P. gingivalis* in NAFLD.

### Indirect Impacts of *P. gingivalis* in NAFLD: Bacteria-Derived Gut Dysbiosis

The effects of *P. gingivalis* on the modulation of gut microbiota have been investigated in many studies. Both intravenous injection and oral administration of the bacteria in a mouse model produced alterations in the gut microbiota (50, 173, 174). Clinical studies have detected remarkably higher proportions of *P. gingivalis* in the guts of NAFLD patients than in those of non-NAFLD controls 99; patients with chronic periodontitis tended to have less diversity in their gut microbiomes (175); the major changes in the gut microbiota of patients with liver cirrhosis result from invasion by oral bacterial species (176). These results indicate a possible relationship between the bacteria, gut, and NAFLD (Figure 2). Studies involving oral administration of *P. gingivalis* to mouse models show that, in contrast to the increased blood endotoxin levels that are typical of gut dysbiosis/barrier dysfunction and lead to systemic inflammation contributing to liver injury, no *P. gingivalis* was detected in the blood system (174), or outgrowth of the bacteria in the gut (80). This strongly supports the speculation that oral *P. gingivalis*-induced endotoxemia-related liver injury indirectly, by inducing gut dysbiosis and barrier dysfunction.

However, despite the evidence that oral *P. gingivalis* can survive in the acidic conditions induced by gastric juice (49), enabling it to enter the gastrointestinal tract, and that dead bacteria from the mouth may stimulate several gut pathogens, upregulate bacterial virulence genes, and thereby increase cytotoxicity (177), the mechanism underlying bacteria-induced gut dysbiosis remains unclear. For example, *P. gingivalis* must inhabit the host cells and trigger immune-inflammatory reactions to initiate subsequent actions, and *in-vitro* studies have shown that intestinal inflammation can be detected following oral administration of *P. gingivalis* (50), but there is as yet no evidence to show whether and how *P. gingivalis* can localize in the gastrointestinal tract; in addition, the bilateral interactions between oral bacteria and gut microbiota should be considered. Furthermore, the lack of large-scale epidemiologic studies and some inconsistent results in different animal models make it difficult to define a clear mechanism underlying the effects of *P. gingivalis* in NAFLD *via* modulation of gut microbiota (93).

On the other hand, *P. gingivalis* is not the only periodontopathogen that can contribute to the pathogenesis of NAFLD. *A. actinomycetemcomitans* can induce gut dysbiosis and impairing glucose metabolism (178); the detection frequency of *T. denticola* in the NAFLD patients was significantly higher than that in the control subjects (99); in patients with liver cirrhosis, more than half (54%) of the patient-enriched, taxonomically assigned bacterial species were of oral origin (mostly veillonella and streptococci) (176, 179). Moreover, in comparison to growing evidence that supports *P. gingivalis* as a cause of gut dysbiosis, the precise mechanism through which *P. gingivalis* exerts its effects has yet to be determined. Further studies are needed to complete our knowledge of the roles of *P. gingivalis* in NAFLD *via* the oral-gut-liver axis.

## Therapeutic Strategies in NAFLD: Targeting Oral Pathogens and Microbial Dysbiosis

**Figure 3:**
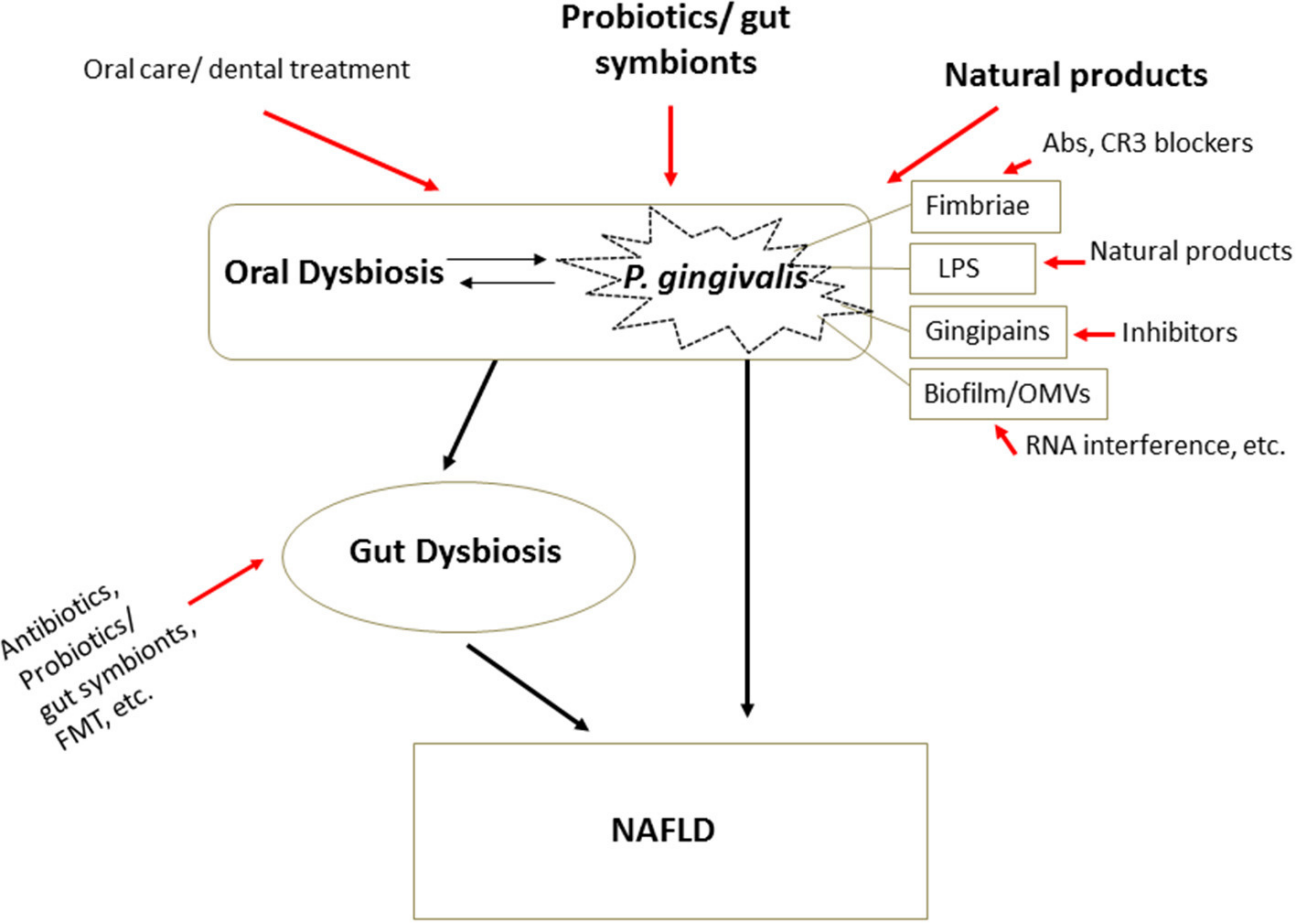
Therapeutic strategies in microbial dysbiosis-related NAFLD. Understanding the mechanism underlying microbial dysbiosis and its impacts on the pathogenesis of NAFLD through the gut-liver, oral-liver, and oral-gut-liver axes may contribute to the development of new options for treatment of the disease. Some very safe options, such as some probiotics, gut symbionts, and natural products, aimed at attenuating oral bacteria and correcting dysbiosis of the oral and gut microbiota, have received substantial attention. Additionally, treatments targeting the structures and virulence factors of *P. gingivalis* offer new treatment options specifically targeting bacteria. Abs, antibodies; FMT, fecal microbiota transplantation.

Dietary factors have been accepted for over a decade as a critical risk factor for NAFLD. Food intake control (caloric restriction) and methods to increase energy consumption, such as physical activity and sports, have therefore been the major treatment options for NAFLD. Vitamin reagents, PPAR agonists, and other treatments are in use or undergoing clinical trials (48). However, most of them are symptomatic treatments, of uncertain efficacy, especially in severe progressive forms such as NASH or HCC. The newly established theory of bacteria-induced NAFLD provides guidance for the prevention, prediction, and treatment of the disease. Attenuating oral bacteria and improving gut dysbiosis are the main therapeutic approaches being studied (Figure 3, Table 3).

**Table 3:** Therapeutic approaches in NAFLD targeting oral pathogens and oral/gut microbial dysbiosis.

**Therapeutic approaches**	**Targets**
Oral care/dental treatment	Oral pathogens & dysbiosis
Natural products	Oral pathogens & dysbiosis, gut dysbiosis
Probiotics/gut symbionts	Oral pathogens & dysbiosis, gut dysbiosis
Prebiotics	Gut dysbiosis
Synbiotics	Gut dysbiosis
FMT	Gut dysbiosis
Abs, CR3 blockers	Fimbriae of *P. gingivalis*
Inhibitors	Gingipains of *P. gingivalis*
RNA interference	Biofilm/OMVs of *P. gingivalis*
Antimicrobial agent-induced membrane vesicles	Biofilm/OMVs of *P. gingivalis*

*Abs, antibodies; FMT, fecal microbiota transplantation*.

### Treatment Based on Controlling Oral Disease

Given the remarkable impacts of periodontal disease in NAFLD, improving oral hygiene, and other approaches targeting pathogenic oral bacteria, are regarded as effective treatment strategies for bacteria-associated NAFLD. Administration of broad-spectrum antibiotics can effectively protect against various liver diseases, and some specific antibiotic treatments have produced improvement in the clinical symptoms of NAFLD, by means of eliminating harmful microbes, lowering circulating endotoxin and transaminase levels, and preventing lipid accumulation in the liver (180). Notably, despite their powerful positive effects, long-term administration of antibiotics may cause toxic side effects, eliciting antibiotic-resistant bacterial strains and itself generating gut dysbiosis (181). There is therefore increasing interest in the safety and specificity of therapeutic approaches targeting pathogenic oral bacteria, such as *P. gingivalis*.

One of the treatment modalities is the administration of probiotics. Two lactic acid bacteria, *Lactococcus lactis* and *Lactobacillus reuteri*, have millennia-long histories of use in the fermentation of foods and are generally accepted as safe for human consumption (“Generally Recognized As Safe,” or GRAS, in US Federal Drug Administration terminology) (182). It has been reported that *L. lactis*, together with one of its derivatives, nisin, was effective in periodontal disease, with potent antibacterial ability but low side effects. Its mechanism involves modulation of the formation, composition, and survival of oral bacterial biofilms (82). Moreover, *L. lactis* treatment reduced hepatic fat accumulation and showed anti-inflammatory effects in the liver, indicating its potential in the treatment of NAFLD (82, 182). Furthermore, antibacterial effects on *P. gingivalis* and other oral pathogens were seen not only from live *L. reuteri* cells but also supernatants from cell-free cultures and heat-killed *L. reuteri* (183), indicating a potent yet safe antibacterial activity of this probiotic.

With regard to the therapeutic approaches specifically targeting the structure of *P. gingivalis*, further methods for inhibiting biofilm formation by modulating OMVs have been investigated, in addition to the biofilm modulation approach mentioned above. These include RNA interference technology, antimicrobial agent-induced membrane vesicles, and some natural products, which have all been reviewed recently (120). Plant-derived products have attracted considerable attention in chronic disease therapy, given their safety. Natural compounds have been demonstrated to inhibit the growth and virulence factor activity of bacteria (184–186). We have reported that carnosic acid, extracted from rosemary plants, exerts potent effects, inhibiting lipid accumulation in both the adipose tissue and liver, improving glucose metabolism and liver functions in the *ob-ob* obesity mouse model (187), and inhibiting lipid accumulation in HepG2 cells (188). Moreover, our studies have clarified that carnosic acid can protect normal hepatocytes from H2O2-induced oxidative stress (189). Interestingly, carnosic acid has been reported to have remarkable antibacterial activities (190, 191). The mechanism is related to its inhibition of biofilm information (192); carnosic acid can also protect against LPS-induced liver injury (193), and is thus a potential new therapeutic approach for treating NAFLD. In our laboratory, we are currently investigating the roles of carnosic acid in NAFLD associated with *P. gingivalis* inoculation in both mouse and cell models.

Anti-fimbrial Ab has also been reported as effective in inhibiting the adherence of *P. gingivalis* to its host cells (194). Inhibition of or pre-immunity against potent virulence factors of *P. gingivalis, s*uch as gingipains, was effective against periodontitis and systemic diseases (195, 196). Moreover, as the CR3 receptor plays an important role in the *P. gingivalis* fimbriae-stimulated adhesion signal, as described above, CR3 attenuation has been considered as an approach for treating periodontitis and *P. gingivalis*-related systemic diseases (197). However, little is known about the significance of the above materials for the management of NAFLD associated with infection by oral pathogenic bacteria such as *P. gingivalis*, which should be investigated further.

### Treatments Targeting Dysbiosis of Microbiota

Since the gut microbiota is closely related to the pathogenesis of NAFLD, various therapeutic approaches targeting gut microbiota have been developed. They include antibiotics, probiotics, prebiotics, synbiotics, and fecal microbiota transplantation, which have recently been reviewed (76, 180). In particular, dietary fiber is cited as useful: it may improve early-stage NAFLD by reducing calorie absorption and correcting the imbalance of gut microbiota (198); *L*. reuteri not only improves the dysbiosis of oral microbiota by targeting biofilm, but also shows the ability to correct gut dysbiosis and protect the liver from inflammatory damage (199). Moreover, a gut symbiont, *Akkermansia muciniphila*, and one of its membrane proteins has produced anti-inflammatory activity and improved gut permeability in animal models (30, 200, 201). Furthermore, human studies have shown that administration of *A. muciniphila* leads to decreased body weight, and the markers associated with inflammation and liver dysfunction in obese objects (202). However, it should be noted that while microbiome-based therapeutic approaches are usually regarded as safe, they cannot be considered risk-free, as they are still bioactive. Therefore, large-scale studies are required to evaluate their safety.

## Conclusions

The close relationship between systemic disease and dysbiosis of both the oral and gut microbiota has been supported by substantial evidence from both basic and clinical studies. In this review, we emphasize one of the most commonly diagnosed such diseases, NAFLD, using it as a typical example of systemic disease, and *P. gingivalis*, which is the major pathogen of periodontitis, and typical of the pathogenic bacteria involved in systemic diseases *via* the oral-liver and oral-gut-liver axes. We focused on the structure-derived functions of *P. gingivalis* in the progression of NAFLD, such as hepatic steatosis, inflammation, and fibrosis, which cause conversion to NASH. NASH is associated with the pathogenesis of HCC, and it is therefore reasonable to suppose that *P. gingivalis* may contribute to HCC by promoting progression to NASH.

Currently, various therapeutic approaches are being developed, targeting *P. gingivalis* and dysbiosis of both oral and gut microbiota. Although most of these remain at the fundamental experimental level, limited to small-scale studies or experiencing difficulties in establishing ideal animal models, improving our understanding of the connections between NAFLD and oral/gut dysbiosis will definitely contribute to achieving the reasonable future goal of treating the disease successfully.

## Author Contributions

TC, TW, TI, and MS conceptualized the review. TW and TI drafted the manuscript. TC and MS edited the manuscript. All the authors read and approved the final manuscript.

## Funding

This study was supported by Grants-in-Aid for Scientific Research (JP 19K07737) from the Japan Society for the Promotion of Science.

## Conflict of Interest

The authors declare that the research was conducted in the absence of any commercial or financial relationships that could be construed as a potential conflict of interest.

## Publisher's Note

All claims expressed in this article are solely those of the authors and do not necessarily represent those of their affiliated organizations, or those of the publisher, the editors and the reviewers. Any product that may be evaluated in this article, or claim that may be made by its manufacturer, is not guaranteed or endorsed by the publisher.
